# Ten areas for ICU clinicians to be aware of to help retain nurses in the ICU

**DOI:** 10.1186/s13054-022-04182-y

**Published:** 2022-10-13

**Authors:** Jean-Louis Vincent, Carole Boulanger, Margo M. C. van Mol, Laura Hawryluck, Elie Azoulay

**Affiliations:** 1 Department of Intensive Care, Erasme Hospital, Université Libre de Bruxelles, Route de Lennik 808, 1070 Brussels, Belgium; 2 Department of Intensive Care, Royal Devon University Healthcare NHS Foundation Trust, Exeter, UK; 3 Department of Intensive Care Adults, Erasmus MC, University Medical Center, Rotterdam, The Netherlands; 4 Critical Care Medicine, University Health Network, University of Toronto, Toronto, ON Canada; 5 Medical Intensive Care Unit, Famirea Study Group, Paris, France

**Keywords:** Burnout, Moral distress, Teamwork, Leadership

## Abstract

Shortage of nurses on the ICU is not a new phenomenon, but has been exacerbated by the COVID-19 pandemic. The underlying reasons are relatively well-recognized, and include excessive workload, moral distress, and perception of inappropriate care, leading to burnout and increased intent to leave, setting up a vicious circle whereby fewer nurses result in increased pressure and stress on those remaining. Nursing shortages impact patient care and quality-of-work life for all ICU staff and efforts should be made by management, nurse leaders, and ICU clinicians to understand and ameliorate the factors that lead nurses to leave. Here, we highlight 10 broad areas that ICU clinicians should be aware of that may improve quality of work-life and thus potentially help with critical care nurse retention.

The coronavirus disease 2019 (COVID-19) crisis has been followed by a serious increase in the shortage of intensive care unit (ICU) personnel, especially nurses, exacerbating a problem that pre-dated the pandemic… and this on a global scale. The reasons are not difficult to elucidate and primarily relate to the prolonged, excessive workload during the pandemic period, often with denial of vacation time; the significant emotional burden associated with the high patient death rate; and the moral distress associated with being unable to provide the usual high quality standard of care, because of the sheer numbers of patients being admitted [1–5]. Personal anxieties around catching the virus and/or passing it on to one’s family, and restrictions and limitations associated with the strict safety measures, including reduced communication and contact with patients and their relatives that resulted in a de-humanizing of care, added to the physical and mental exhaustion felt by healthcare staff [1–4, 6, 7]. In some hospitals, non-critical care-trained nurses or doctors were transferred to the ICU to fill nursing staff gaps, in ways that created a feeling that the skill set and knowledge of trained critical care nurses was devalued and that they could be easily replaced. In addition, at the beginning of the pandemic, there were visible signs of recognition and appreciation by the public for the extra work—indeed, in many countries, people came out of their homes to applaud the medical and nursing personnel at the same time every day—but this initial mark of gratitude soon lapsed and was replaced by complaints, threats from COVID deniers, protests, attacks on social media, and even legal claims by patients and especially their families, which certainly did not help improve morale.

As a profession already associated with high rates of stress and burnout [8–10], the additional pressures associated with the pandemic have led many nurses, especially those working on acute units with large numbers of COVID-19 patients, to become even more disillusioned so that many have left the ICU to work in other Departments and, worryingly, some have left the profession completely. Adequate staffing is recognized as being vital to ensure good patient care and, consequentially, outcomes [11–13]. There is therefore an urgent and ongoing need to cultivate and support strategies that help make critical care nursing attractive and reduce the pressures and stresses faced by nurses, to ensure that sufficient numbers of well-trained nurses are present on our units. Increasing wages may seem an obvious solution; indeed, in many countries, critical care nurses are paid at the same level as nurses without any additional specialized training, yet the responsibilities of critical care nurses are much greater. However, while financial incentives are necessary and may indeed help attract staff in the short-term, they will not be sufficient to retain staff if other aspects to improve job satisfaction are not tackled simultaneously; adequate pay must be seen as just one component of a suite of solutions [14, 15].

Here we discuss—in no specific order of importance—10 broad areas that ICU clinicians should be aware of which may improve quality of work-life and thus potentially help with critical care nurse retention (Table 1). Of note, although the focus of this commentary is on the recruitment and retention of critical care nurses, burnout is significant among all members of the ICU team [16, 17] and many of the aspects we propose here to help in the retention of critical care nurses may equally apply to the retention of other team members.

- *Recognition, respect, and value* If the COVID-19 pandemic has taught us nothing else, it has highlighted the incredible worth and importance of critical care nurses. They are highly trained professionals, with specialized knowledge, and skills honed with every patient cared for. Such qualities are not replaceable; not everyone can perform the role of a critical care nurse. Non-critical care nurses and other healthcare providers may provide significant help and can relieve critical care nurses from performing tasks that are not ICU skill specific (e.g., patient hygiene, turning, suctioning), but how such personnel are recruited and deployed needs careful consideration for people to feel valued and supported. Such assistance may permit critical care nurses to really focus on what they trained for: critical thinking, crisis management, and situational awareness in caring for those with life-threatening illnesses. It is important to recognize and appreciate the important role of critical care nurses as skilled members of the ICU team, to thank them, and to see the person in the professional to make them feel they are valued colleagues. A lot of attention has been paid to humanizing medicine for the patients (and their families) that we treat. It is past time for attention to be paid to humanizing it for healthcare providers, especially those working in critical care. This may seem obvious and even unnecessary, but such acknowledgement is all too frequently overlooked—taken as understood among professionals—and yet is crucial for maintaining morale, decreasing stress and promoting a healthy, safe, and high performance workplace. Understanding and valuing the contribution of all ICU team members, including nursing staff, fosters a team approach to the significant difficulties faced.
- *Role and responsibility* Within the ICU, each member of staff should have some responsibility, relative to his/her qualifications, interests, and experience. Nurses have knowledge and competences specific to their own professional domain and should take the lead in these aspects of ICU patient care. However, the degree of allowed responsibility varies considerably internationally. In most countries, nurses can, for example, initiate fluid challenges [18], perform electrolyte replacement, or titrate vasopressor agents while monitoring and assessing the patient’s response. They should be involved in checking sedation and analgesia levels are appropriate, adjusting feeding, and other essential aspects of patient management, helpfully summarized in the FAST-HUG mnemonic [19]. Within defined boundaries, which may differ in different units and in different countries, and with adequate support, nursing staff should be encouraged to use their clinical judgement and act on their own. These aspects should be discussed within each ICU team to determine which tasks nurses can perform and which skills nurses could be taught to achieve in the future to promote engagement, career development, and patient care. ICU teams should also discuss and share their experiences as a way of mentoring and supporting change, recognition, and promotion of the evolving critical care nursing role.
- *Intellectual stimulation and professional development* It is well recognized that intellectual stimulation and feelings of self-accomplishment are an essential component of job satisfaction across professions. Each nurse should be able, and indeed encouraged and mentored, to develop expertise in one or several specific aspects of patient management, if they wish to do so. They could become the recommended contact for any question related to issues including but not limited to wound care, optimal feeding, renal replacement therapy, a continuous positive airway pressure (CPAP) system, a new piece of monitoring equipment, appropriate sedation, family liaison, and person-centered care. They could also participate in or be a designated resource for specific research trials if a research nurse is not readily available. Awareness of who holds these roles and valuing the associated expertise and input supports personal development and the sense of worth. A higher level of autonomy, leadership and broadening of the critical care nursing profession offers career-enhancing possibilities that may lead to reduced work-related stress and potentially help retain more nurses in our profession [20]. In hospitals where this applies, nurses should be provided with opportunities and encouragement for promotion within the ICU or outside the ICU borders, whether as an ICU representative or as a role model for others of what a career in intensive care can lead to.
- *Teaching opportunities* Nurses of all levels should be given the opportunity to teach in their area(s) of expertise, not only to other nurses, but also to doctors (trainees and attending staff) and other allied healthcare professionals. Encouraging and supporting nurses to present at unit/team seminars, to participate in simulation, case-based teaching, gamification, to lead teaching on new policies, new equipment and new practices, and to present results of emerging research in ICU- or hospital-wide journal clubs or even at (inter)national congresses is another way to engage nurses and send a message to the entire ICU team, healthcare organization, and even ministries of health that the nurses are an integral and highly valued and respected part of the ICU team.
- *Good leadership and management* Dynamic, motivated leaders and managers are important to provide a good example of what is possible and specific leadership patterns are linked to higher levels of job satisfaction among nursing staff [21]. Head nurses should be offered regular leadership and mentorship training and encouraged to listen to the concerns of their team members, to provide positive, constructive feedback rather than negative criticism, and to understand the career goals of their nursing staff while seeking opportunities to help fulfill these goals. Since critical care medicine is a collaborative specialty, it is also important for critical care physicians to consider how they too may help foster nurses at all stages of their career to achieve their goals, open doors to new opportunities, and provide mentorship. Such actions would make the ICU a supportive, encouraging environment, and facilitate both recruitment and retention.
- *Team work/collaborative practice* Nurses are fundamental members of the ICU team and should be encouraged to actively contribute during clinical rounds and to other discussions regarding patient management; after all, they usually spend more time with the patient than the medical staff. Indeed, a team approach is essential for any professional activity, but is even more important in our discipline—people’s lives can literally depend on every team member feeling safe to raise concerns and/or share their perceptions of responses to treatments. ICUs must foster an inclusive, non-intimidating, collaborative work environment in which the contributions and opinions of all team members are valued.
- *Clinical discussion and exchange* Although regular, planned formal discussions of clinical cases that engage key educational principles are valuable, impromptu, informal discussions of individual patients or relevant topics are also important. Nurses should feel empowered to initiate such discussions with other members of staff and to speak openly as equals. Indeed, all members of the team must feel they can raise any issue with their colleagues and that their questions and opinions are relevant and appreciated. Open dialogue with the nurse at the bedside promotes effective communication in the interests of patient care. Ideas from nursing staff to improve patient care, patient safety, and the functioning of the ICU should be listened to and actioned when possible and appropriate. Taking time to share concerns can help to ease the burden of care and ensure broad understanding, thereby reducing the risk of conflict within a team that can occur as a result of ineffective or absent communication.
- *Good work-life balance*/*wellness/rehumanizing the workplace* The ICU is a very fast paced, challenging workplace environment and efforts to meet patient needs are often overwhelming. Given their firsthand knowledge of current workload, nurses can provide important input into decisions regarding admission triage of referrals or transfers from outside facilities, and what is possible to maintain patient safety and quality of care. Furthermore, when new tasks, such as implementation of new policies, procedures or equipment, are required, critical care professionals should ensure that the responsibilities are shared as much as possible and that one group, e.g., nurses, respiratory therapists,… are not disproportionately burdened. The same applies to the initiation of new research projects within the ICU environment. In addition, everyone needs time off, to relax and recuperate both mentally and physically, and to enjoy family life and leisure time. This is easy to say, but not so easy to put into action. However, small steps can help, such as ensuring sufficient rest periods between shifts; providing work schedules well ahead of time as much as possible; accepting that nurses can say no to staying late/working overtime; and respecting family and personal commitments and responsibilities. Finally, although part-time jobs have not been encouraged and in some places are not currently available for nurses, especially in an acute care setting, the flexibility they offer could play an important role in encouraging some nurses to stay. Wellness is not just a personal responsibility but needs to be incorporated into our work environment in practical ways to decrease stress and strain.
- *Psychological support* The ICU is often an environment of life-death situations, and constantly caring for high-burden, critically ill patients can create considerable emotional and mental stress. Team debriefings after particularly difficult or distressing cases are important, but individual support from colleagues seems to be most valued and appropriate among nurses [7]. For example, WhatsApp groups during the COVID-19 pandemic worked well. Sometimes professional help is needed and should be freely available to all at all times, without stigmatizing those who do (or do not) make use of it. The expenses for such support will be more than compensated for by the positive effects on nurse morale and thus workflow. Resilience training, or similar programs to help manage stress, may also be beneficial [22].
- *Humane care* Nursing activities are not limited to monitoring, feeding, and medication administration, but include providing continuing psychological support of the patients, and often their families. A perception that patients are not being cared for humanely or that care given is inappropriate [5, 23] can promote disillusion and frustration. End-of-life care, in particular, is not always as good as it could or should be for ICU patients. Conflict in end-of-life decision making is associated with moral distress for critical care nurses [24]. Nurses should feel able to raise the need for a possible end-of-life decision with other members of staff if they feel it is relevant for a particular patient. Listening to their ideas for improvement and discussing and developing inclusive quality improvement initiatives can help. Nurse facilitators are one option to help improve end-of-life communication among staff members, the patient and their family [25].

**Table 1:** Some key areas for clinicians to consider to help keep nurses on the ICU

*Recognition, respect, and value*
Acknowledgment of important role; recognition of high levels of training, knowledge and skills, situational awareness and crisis management skills, and personal qualities and commitment/ dedication as individuals
*Role and responsibility*
Recognition of responsibilities in complex patient resuscitation and management, recognition of deterioration and patient safety events,involvement in complex decision-making
*Intellectual stimulation and professional development*
Encouragement, mentorship, and support in development of new knowledge, skills career opportunities and growth
*Teaching opportunities*
Encourage leadership role, mentor and support while creating teaching opportunities to other nurses, doctors…, at (inter)national meetings
*Good leadership and management*
Positive, constructive feedback to encourage development of new expertise, promote engagement and encourage in quality of care, patient safety and research
*Team work/collaborative practice*
Active involvement within team, participation in unit activities
*Clinical discussion and exchange*
Sharing, initiating education opportunities and formal/informal open discussions about pathophysiology of illnesses, patient care, and safety
*Good work-life balance/wellness/rehumanizing the workplace*
Explore, discuss acceptable working hours (part-time?), ensure supportive team structures to promote and allow safe workloads, adequate breaks and opportunities for self care
*Psychological support*
Normalize mental health impact of care, promote team debriefings and individual support, promote collegial support, promote access to professional support, access to wellness initiatives
*Humane care*
Promote recognition of the person in patient and family care, promote participation in end-of-life discussions and team collaboration to understand the uniqueness of each situation/its impact on all involved and implications on communication and how the end-of-life should be approached to convey respect for patients/families in decision-making on treatment goals and limits, and during withholding/withdrawing of life-sustaining treatment or palliative care

The current nurse shortages in the ICU are not new, but have been amplified by the COVID-19 pandemic. It is more than time to acknowledge the need for change, at all levels of the healthcare system from the individual to the institutional to the broader governmental and societal level [26]. Insufficient nurse numbers impact not only patient safety and outcomes [11–13], but also create a vicious cycle—fewer nurses lead to increased workload, so there is greater pressure on remaining staff, leading to increased stress and burnout, which in turn leads to increased numbers of nurses leaving the specialty, thereby further increasing the workload. Adequate nurse staffing is thus of prime importance not only for hospital managers and nurse leaders, but for all members of the ICU team, including doctors. Having sufficient numbers of nursing staff with high job satisfaction will improve the quality of care delivered to patients and the quality of work-life for all on the ICU. Understanding and valuing the contribution of all ICU team members, including nursing staff, fosters a team approach to the significant difficulties faced. Awareness of ways in which medical staff can contribute to supporting their nursing colleagues ensures a team approach to the challenges of the fallout from the pandemic on the background of an already stretched nursing team.

The elements discussed herein may not be applicable or relevant to all hospitals or units but can serve as a general framework for further discussion to help understand why critical care nurses leave the profession and what strategies can be actively established and supported at a local level to encourage them to stay.

## Data Availability

Not applicable.

## Abbreviations

COVID-19: Coronavirus disease 2019
CPAP: Continuous positive airway pressure
ICU: Intensive care unit
